# Editorial: The evolution in pharmacology of infectious diseases: 2022

**DOI:** 10.3389/fphar.2024.1386077

**Published:** 2024-03-12

**Authors:** Exequiel O. J. Porta, Ali Saffaei, Karunakaran Kalesh

**Affiliations:** ^1^ UCL School of Pharmacy, University College London, London, United Kingdom; ^2^ Department of Clinical Pharmacy, School of Pharmacy, Shahid Beheshti University of Medical Sciences, Tehran, Iran; ^3^ School of Health and Life Sciences, Teesside University, Middlesbrough, United Kingdom

**Keywords:** Chagas disease, COVID-19, cystic fibrosis, hepatitis C, infectious diseases, leishmaniasis, pharmacology, tuberculosis

## 1 The landscape of infectious diseases post-pandemic: a pharmacological perspective

The era post-2020 has marked a defining moment in the evolution of pharmacology, with a particular emphasis on infectious diseases (Baker et al., 2022). This period has been characterized by the relentless global campaign against COVID-19 and the emergence of a spectrum of other infectious pathogens (Tabish, 2020). It was during this time that the field of pharmacology experienced an unprecedented amalgamation of global research endeavors and the development of innovative therapeutic solutions. The research conducted in these years has not only paved the way for groundbreaking therapeutic strategies but has also illustrated the extraordinary capacity for resilience and innovation within the scientific community (Rijs and Fenter, 2020). These advancements, born out of necessity in the face of global health crises (Betz et al., 2023), have reshaped the landscape of infectious disease treatment and prevention.

This editorial offers an insightful examination and analysis of the significant advancements in the field of infectious diseases from 2022 to 2023, specifically focusing on how these developments have transformed our approaches to combating such diseases. At the heart of this exploration is an overview of 15 pivotal articles featured in our Research Topic. These articles, contributed by 120 distinguished experts worldwide, represent a critical blend of knowledge, combining a range of expert insights and discoveries. This collective wisdom is crucial for developing innovative strategies to treat infectious diseases and significantly enhances our methods for addressing global health challenges. Additionally, this editorial delves into the interplay between pathogens and recent advances in drug development, highlighting how this period has served as both a rigorous test of scientific resolve and a catalyst for notable progress in medical science. By exploring the dynamic relationship between emerging diseases and pharmacological innovations, it underscores how this era has been challenging yet instrumental in driving substantial advancements in the evolution of the medical field.

## 2 COVID-19 therapeutic developments: key research findings and innovations

In the ongoing struggle against COVID-19, the scientific community has been unwavering in its quest to uncover new therapeutic avenues. Umakanthan et al. made a significant contribution with their study on the unexpected benefits of Statins, commonly used for lipid management, in reducing mortality among hospitalized COVID-19 patients. This groundbreaking finding not only highlights the anti-inflammatory and immunomodulatory properties of statins but also opens new avenues for COVID-19 treatment, suggesting a broader application of these drugs beyond their traditional use. Complementing this, Barati et al.’s research on the combination of Noscapine and Licorice for cough relief in COVID-19 outpatients indicated a slight superiority over traditional treatments. The study is particularly pertinent as it addresses the symptomatic burden experienced by COVID-19 patients and emphasizes the importance of effective symptom management strategies in viral infections. It signifies the potential of integrating traditional remedies with modern pharmacological practices, a theme that resonates throughout our Research Topic. For instance, Maen et al.’s study on Thymoquinone formulation (NP-101) against SARS-CoV-2 revealed its potential as a novel treatment, pointing to new directions in antiviral therapy. This finding not only contributes to the ongoing efforts against COVID-19 but also underscores the importance of exploring natural compounds in drug development.

The exploration of combination therapy as a strategy for treating COVID-19 has gained traction, as highlighted by Akinbodale et al. (2022). This approach involves the concurrent use of multiple medications, targeting the virus from various perspectives to potentially enhance patient outcomes. A notable study by Dastan et al. examined the combined use of Tocilizumab, a biologic drug approved for treating moderate to severe rheumatoid arthritis in adults, and Baricitinib, an immunomodulatory medication for rheumatoid arthritis. Their research, focusing on severe COVID-19 cases, revealed that while this combination did not significantly reduce mortality rates, it was associated with a reduced necessity for Intensive Care Unit (ICU) admission. This outcome underscores the potential benefits of such combination therapies in certain patient groups and clinical settings. It also emphasizes the growing importance of personalized medicine in effectively managing COVID-19.

In a real-world study, Zhong et al. evaluated the efficacy and safety of Nirmatrelvir/Ritonavir co-administration in patients with rheumatic disease infected with SARS-CoV-2. This study found that using Nirmatrelvir/Ritonavir as part of standard or early treatment regimens led to a shorter time for symptom resolution compared to a control group. Even when this combination of drugs was administered after 5 days of symptom onset, it still offered benefits for rheumatic patients. The study highlighted the importance of early Nirmatrelvir/Ritonavir utilization and following the recommended regimen, showing favorable outcomes and an acceptable safety profile for immunosuppressed rheumatic patients. In addition, Wei et al.’s comparative analysis of Azvudine, originally used for HIV-1, and Nirmatrelvir/Ritonavir concluded that Azvudine offered similar safety but slightly better clinical benefits in hospitalized patients. This finding adds to the growing body of literature on effective COVID-19 treatments and highlights the importance of continual evaluation and comparison of therapeutic options in the rapidly evolving landscape of the pandemic. In a similar vein, Zhu’s perspective on Azvudine, showcased its effectiveness in treating moderate COVID-19 cases. This work exemplifies the potential of drug repurposing in the pandemic era, providing a cost-effective solution to the global health crisis. It also underscores the importance of adaptability in pharmaceutical research, as existing drugs can be re-evaluated and repurposed to meet emerging health challenges.

Finally, the investigation into the cardiac impacts of Remdesivir by Hajimoradi et al. shed light on the complexities of COVID-19 treatment, particularly the incidence of sinus bradycardia in patients treated with this antiviral drug. This finding highlights the need for comprehensive monitoring during treatment, underscoring the multifaceted nature of COVID-19 and the importance of considering the broader implications of antiviral therapies.

## 3 Innovations beyond COVID-19

Moving beyond COVID-19, these years also witnessed significant advancements in the treatment of other infectious diseases. For instance, El-Mahdy et al.’s study on chronic hepatitis C patients treated with direct-acting antivirals showed marked improvements in liver function and antioxidant profiles. This work marks a milestone in the management of this chronic infection and highlights the progress made in understanding and treating hepatitis C.

In the realm of cystic fibrosis, a debilitating genetic disease, Rakhshan et al.’s evaluation of inhaled Amikacin as an adjunct therapy in treating *Pseudomonas aeruginosa* exacerbations represents a significant step forward. This clinical trial not only highlights innovation in treatment strategies but also emphasizes the importance of addressing the specific needs of patients with cystic fibrosis.

Drug repurposing continued to be a key theme in the research landscape, as exemplified by Porta et al.’s review on Chagas disease. This neglected tropical disease, caused by the protozoan parasite *Trypanosoma cruzi*, has been a longstanding public health challenge, particularly in Latin America. The review emphasized the urgent need for new treatment strategies and highlighted the potential of repurposing existing drugs as a cost-effective and expedient approach to address this issue. In the field of cutaneous leishmaniasis, another neglected tropical disease, Hakamifard et al.’s clinical trial on liposomal Clarithromycin, an antibiotic, combined with the antileishmanial agent Glucantime suggested a significant effect in reducing lesion size. This clinical trial is particularly relevant for regions where leishmaniasis remains a significant public health issue and where effective treatment options are sorely needed.

The challenge of drug resistance, a critical concern in modern medicine, was highlighted in Sichen et al.’s analysis of multidrug resistance in tuberculosis patients in Northeast China. This report underscores the need for continued vigilance and innovation in the fight against drug-resistant infections, a challenge that continues to evolve and requires new strategies. Similarly, Wang et al.’s review on the complexities of non-tuberculosis mycobacteria skin infections called for a more nuanced understanding and tailored therapeutic strategies. This review reflects the evolving nature of infectious diseases and the corresponding need for adaptive and targeted treatment approaches.

In veterinary medicine, Lee et al.’s pharmacokinetic/pharmacodynamic study on Tylosin, a macrolide antibiotic, in pigs co-infected with *Actinobacillus pleuropneumoniae* and *Pasteurella multocida* provided valuable insights into effective dosing strategies. This study not only advances our understanding of antimicrobial therapy in animal health but also reflects the interconnectedness of human and animal health, a concept integral to the One Health approach (Mackenzie and Jeggo, 2019).

## 4 Conclusion

The period from 2022 to 2023 in pharmacology has been characterized by a blend of innovation, repurposing, and deepened understanding of infectious diseases. The articles in our Research Topic have significantly contributed to this evolving landscape, where old and new therapies converge, providing hope and direction for future research and patient care. This era’s developments serve as a beacon of progress, illuminating the path towards more effective, safe, and accessible treatments for infectious diseases globally. As we move forward, the lessons learned, and strategies developed during this time will undoubtedly continue to influence the field of pharmacology and the broader scientific community’s approach to addressing health challenges.
